# Biphasic Effects of Blue Light Irradiation on Human Umbilical Vein Endothelial Cells

**DOI:** 10.3390/biomedicines9070829

**Published:** 2021-07-16

**Authors:** Kejia Kan, Yifei Mu, Marielle Bouschbacher, Carsten Sticht, Natalia Kuch, Martin Sigl, Nuh Rahbari, Norbert Gretz, Prama Pallavi, Michael Keese

**Affiliations:** 1 Department of Vascular Surgery, Medical Faculty Mannheim, Heidelberg University, 68167 Mannheim, Germany; Kejia.Kan@medma.uni-heidelberg.de (K.K.); Yifei.Mu@medma.uni-heidelberg.de (Y.M.); Natalia.Kuch@medma.uni-heidelberg.de (N.K.); 2 European Center of Angioscience ECAS, Medical Faculty Mannheim, Heidelberg University, 68167 Mannheim, Germany; 3 Urgo Research Innovation and Development, 21300 Chenôve, France; m.bouschbacher@fr.urgo.com; 4 NGS Core Facility, Medical Faculty Mannheim, Heidelberg University, 68167 Mannheim, Germany; Carsten.Sticht@medma.uni-heidelberg.de; 5 First Department of Medicine, Medical Faculty Mannheim, Heidelberg University, 68167 Mannheim, Germany; martin.sigl@umm.de; 6 Department of Surgery, Medical Faculty Mannheim, Heidelberg University, 68167 Mannheim, Germany; Nuh.Rahbari@umm.de; 7 Medical Research Centre, Medical Faculty Mannheim, Heidelberg University, 68167 Mannheim, Germany; Norbert.gretz@medma.uni-heidelberg.de

**Keywords:** blue light, photobiomodulation, endothelial cell, angiogenesis, apoptosis, reactive oxygen species

## Abstract

Blue light regulates biological function in various cells, such as proliferation, oxidative stress, and cell death. We employed blue light illumination on human umbilical vein endothelial cells utilizing a LED device at 453 nm wavelength and revealed a novel biphasic response on human umbilical vein endothelial cells (HUVECs). The results showed that low fluence blue light irradiation promoted the fundamental cell activities, including cell viability, migration and angiogenesis by activating the angiogenic pathways such as the VEGF signaling pathway. In contrast, high fluence illumination caused the opposite effect on those activities by upregulating pro-apoptotic signaling cascades like ferroptosis, necroptosis and the p53 signaling pathways. Our results provide an underlying insight into photobiomodulation by blue light and may help to implement potential treatment strategies for treating angiogenesis-dependent diseases.

## 1. Introduction

Endothelial cells (ECs) constitute the blood vessel’s innermost [1,2,3] lining and play a critical role in maintaining the homeostasis of blood vessels. Angiogenesis, a physiological process of new vessel formation from existing vessels, depends on various functions of ECs. It is a complex multistep activity that occurs physiologically in embryonic development, organogenesis and wound healing [1]. It also plays a role in pathological states including carcinogenesis, diabetic retinopathy and chronic polyarthritis [1].

During angiogenesis, the vascular basement membrane is degraded by proteolytic enzymes secreted by ECs to allow migration of ECs through the basement membrane to the perivascular matrix [2]. After that, ECs proliferate, adhere, and connect to each other to form tube-like structures. Then, the perivascular matrix is remodeled, and the ECs are surrounded by vascular smooth muscle cells. A vascular anastomosis is formed with the newly formed vascular networks [2]. These steps are well-regulated by a multitude of cytokines, the extracellular matrix and proteolytic enzymes [2]. Any imbalance of the above pro-angiogenic or anti-angiogenic factors would lead to the dysregulation of angiogenesis and initiate the progression of diseases [3,4]. Treatments targeting ECs could be a potential therapeutic approach for angiogenesis-dependent diseases.

Blue light is one component of visible light with wavelength ranges from 380 nm to 500 nm. Due to its high energy, blue light has displayed bactericidal potency similar to ultraviolet (UV) light [5]. Moreover, blue light was proved for its anti-inflammatory properties, which are helpful for the treatment of acne vulgaris [6,7] and psoriasis [8]. Other conditions like neonatal jaundice [9] and local pain [10] are also treated by blue light.

The role of the blue light on ECs or angiogenesis remains less well defined. Several in vivo studies indicated a potential application of blue light in angiogenesis. One animal study showed that blue light (470 nm) treatment could increase angiogenesis and promote wound healing in an ischemia rodent flap model. The ischemic tissue necrosis was reduced, and flaps showed a significantly increased tissue perfusion at day 7 in the blue light-emitting diode (LED)-treated group [11]. Another clinical study showed that blue light exposure (453 nm) could decrease blood pressure by increasing circulating nitric oxide thereby improving the EC function [12].

In the view of contradictory findings, blue light (475 nm) did not affect the migration of ECs or vasculogenesis [13] and the growth of aortic ECs was inhibited after blue light treatment (425–500 nm) [14]. Thus, we here aimed to systematically investigate the effects of blue light (453 nm) on EC cellular parameters such as viability, proliferation, migration, and angiogenic functions.

## 2. Materials and Methods

### 2.1. Human Umbilical Vein Endothelial Cells Isolation and Cell Culture

Human umbilical vein endothelial cells (HUVECs) were isolated from human umbilical cords using the methods described by Jaffe et al. [15]. Umbilical cords were obtained from donors from Gynecology and Obstetrics department. HUVEC isolation was approved by the local ethics committee (Medizinische Ethik-Kommission II, Medizinische Fakultät Mannheim, 2015-581N-MA, Mannheim, Germany). Isolated endothelial cells were maintained at 37 °C under 5% CO_2_ in endothelial cell growth medium (Provitro, Berlin, Germany) supplemented with 5% FBS(Gibco, Brasilia, Brazil), endothelial cell growth supplement mix (Provitro, Berlin, Germany) and 1% antibiotics (100 units/mL penicillin-streptomycin) (Provitro, Berlin, Germany). HUVECs from three donors were pooled and used in experiments between passages two to five.

### 2.2. Blue Light Irradiation

The blue light LED device developed by URGO RID (Chenôve, France) within the EU project MEDILIGHT with a peak wavelength of 453 nm was used in this study. The irradiation fluence was quantified using the power meter 843-R-USB from Newport Corporation (Newport, Irvine, AB, Canada). A cycling irradiation mode of 30 s on and 30 s off was used to avoid overheating.

### 2.3. Cell Viability Assay

Cell viability was determined using the colorimetric cell viability kit III (XTT) (PromoCell, Heidelberg, Germany). HUVECs (1 × 10^4^ cells/well) were seeded in 96-well black plates (Corning Incorporated, Corning, NY, USA) one day before irradiation (80% cell confluence). XTT assay was performed as per the manufacturer’s information 24 h post-irradiation. Results were normalized to the non-irradiated controls.

### 2.4. Cell Proliferation Assay

The colorimetric ELISA-BrdU kit (Sigma-Aldrich, Munich, Germany) was used to determine the blue light effect on cell proliferation. Per well, 1 × 10^4^ of HUVECs were seeded in a 96-well black plate (Corning Incorporated, Corning, NY, USA). A cell confluence of 80% was reached before irradiation. The BrdU assay was performed 24 h after irradiation as described in the users’ manual. Results were normalized to the non-irradiated controls.

### 2.5. Reactive Oxygen Species Measurements

#### 2.5.1. Measurement Using The Redox Sensor

Grx1-roGFP3 is a ratio-metric molecular fluorescence sensor that dynamically tracks the glutathione redox potential with high sensitivity within the living cells. The sequence of Grx1-roGFP3 was a kind gift from Dr. Manfred Frey (Steinbeis-Innovationszentrum Zellkulturtechnik, University of Applied Sciences, Mannheim, Germany). Grx1-roGFP3 was synthesized via GENWIZ service from Sigma Aldrich and cloned into pHR’SIN-cPPT-SEW [16] via restriction sites BamHI and XbaI. Lentivirus particles were produced as previously described [17] and HUVECs were transduced. Briefly, a stock of pHR’SINGrx1-roGFP3 was prepared and stored at −80 °C. Various dilution of the viral stock was used for transduction of HUVECs seeded in a 24-well plate (Corning Incorporated, Corning, NY, USA). Transduction efficiency was determined by fluorescent microscopy. With 1:10 dilution of the viral stock 95% transduction efficiency was observed and was used in all the experiments. The stability of the vector over passages was verified by qualitative assessment of GFP under the fluorescence microscope. Up to three passages after transduction, no evident GFP expression changes were observed.

1 × 10^4^ cells/well Grx1-roGFP3 transduced HUVECs were seeded in the 96-well black plate (Corning Incorporated, Corning, NY, USA) reaching 80% confluence and treated with blue light. The fluorescence intensity in each well was detected at an excitation wavelength of 395 nm and 485 nm and an emission wavelength of 550 nm by using a microplate reader (Tecan, Männedorf, Switzerland). The ratio of emission (395 nm/485 nm) was calculated and plotted over time. The oxidation of the roGFP3-Grx redox sensor resulted in an increase in the emission fluorescence at 528 nm when excited at 485 nm and a decrease in emission fluorescence when excited at 395 nm.

#### 2.5.2. Measurement Using the 2′,7′-Dichlor-dihydrofluorescein-diacetate (DCFH-DA) Dye

The production of intracellular reactive oxygen species (ROS) was determined using DCFH-DA assay (Sigma-Aldrich, Munich, Germany). Per well, 1 × 10^4^ HUVECs were seeded into the 96-well black plate and incubated overnight at 37 °C until 80% confluence. After irradiation, the cells were incubated with 10 μM DCFH-DA in a serum-free medium for 30 min in the dark. Afterward, the plates were rinsed with PBS thrice to remove the free DCFH-DA. The fluorescence intensities were detected at an excitation wavelength of 488 nm and an emission wavelength of 525 nm using a microplate reader (Tecan, Männedorf, Switzerland). Results are shown as a percentage as compared to non-irradiated cells.

### 2.6. Apoptosis Assay

Apoptosis was assessed using FITC Annexin V and Propidium iodide (PI) kit (BioLegend, San Diego, CA, USA). The assay was performed according to the manufacturer’s instructions. HUVECs were harvested 24 h after blue light irradiation, stained with FITC Annexin V and PI, and analyzed using a BD FACSCalibur flow cytometer (BD Biosciences, Heidelberg, Germany). Cells with Annexin V+ and Annexin V+/PI+ were defined as apoptotic cells.

### 2.7. Migration Assays

#### 2.7.1. Wound Healing Assay

5 × 10^4^ HUVECs per well were seeded in six-well plates (Corning Incorporated, Corning, NY, USA) and grown to confluence. Then the cells were scratched across the surface of the well by a 20 µL pipette. The detached cells were removed by washing twice with PBS. After that, cells were treated with blue light. Images were taken at different time points (0, 6 and 9 h) and analyzed with ImageJ software (version 1.51s, NIH Image, NIH, Bethesda, MD, USA). Wound closure was determined by the equation as follows:

Wound closure (%) = (Original wound area—area at each time point)/Original wound area.

#### 2.7.2. Transwell Assay

8 × 10^4^ HUVECs were seeded in each transwell insert (Corning Incorporated, Corning, NY, USA), pre-coated with 1% gelatin and treated with blue light. After 6 h [18,19,20], the cells in the inserts were fixed with 4% paraformaldehyde for 30 min, washed with PBS two times and stained with 0.1% Crystal violet for 30 min. Five representative fields were randomly chosen, and the numbers of penetrated cells were counted using ImageJ software (version 1.51s, NIH Image, NIH, Bethesda, MD, USA).

### 2.8. Angiogenic Assays

#### 2.8.1. Tube Formation Assay

5 × 10^3^ HUVECs were seeded per well in the 15-well μ-angiogenesis slides (Ibidi GmbH, Munich, Germany) and pre-coated with reduced growth factor Matrigel (Corning Incorporated, Corning, NY, USA). Then the cells were treated with blue light. Tube-like structures were imaged with an inverted Microscope (Leica Mikroskopie & Systeme, Wetzlar, Germany) after 6 h incubation. Total tube length of five random fields was quantified with “Angiogenesis Analyzer” for ImageJ software (version 1.51s, NIH Image, NIH, Bethesda, MD, USA) [21].

#### 2.8.2. Spheroid Sprouting Assay

GFP-positive HUVEC spheroids were prepared using the hanging drop method [22]. These spheroids were transferred into 15-well μ-angiogenesis slides (Ibidi GmbH, Munich, Germany) and pre-coated with 5 µg/mL collagen I from rat tail (Corning Incorporated, Corning, NY, USA). Then cells were treated with blue light. After 8 h, the sprouting was stopped by adding amplifying hydrogel solution (AHS). Table 1 shows the AHS composition. The slides were stored at 4 °C overnight. The next day, after the polymerization of the AHS solution the spheroids-containing gel was taken out for imaging. All the samples were imaged using a Leica SP8 laser confocal fluorescence microscope (Leica Mikroskopie & Systeme, Wetzlar, Germany) with the excitation wavelength at 488 nm and emission wavelength at 509 nm. The sprouting area of each sample was measured using ImageJ software (version 1.51s, NIH Image, NIH, Bethesda, MD, USA).

### 2.9. RNA Isolation and Sequencing

Total RNA was extracted using AllPrep DNA/RNA/Protein Mini Kit (Qiagen, Hilden, Germany) according to the manufacturer’s instructions. RNA was quantified using the Spark microplate reader (Tecan, Männedorf, Switzerland) and quality was checked with the Agilent 2100 Bioanalyzer and the RNA 6000 Nano Kit (Agilent, Waldbronn, Germany). Samples with an RNA integrity number (RIN) above 9.5 were used for RNA sequencing. The sequencing was performed by BGI Tech Solutions Co. (Hong Kong, China).

## 3. Bioinformatic Analysis of RNA Sequencing

To analyze the RNA sequencing data, the R software (version 3.6.3) and NGS analysis R package systemPipeR (version 1.22.0) were used. Quality control and adapter trimming of raw data were performed using FastQC (version 0.11.5) and Trim-galore (version 0.4.1) (www.bioinformatics.babraham.ac.uk, accessed on 27 April 2020). The resulting reads were then aligned to the reference human genome GRCh38.p13 and counted using Kallisto (version 0.46.1). The count data were transformed to log2-counts per million (logCPM) with the limma package (version 3.44.3). Differential gene expression analysis was performed using the DESeq2 package (version 1.28.1). Gene enrichment analysis was made with the fgsea package (version 1.14.0) and the EnrichmentBrowser package (version 2.18.1) using the public pathway database KEGG (https://www.genome.jp/kegg/pathway.html, accessed on 27 April 2020).

### 3.1. Real-time Quantitative PCR

Total RNA was isolated as described above. cDNA was synthesized by reverse transcription of the total 500 ng RNA with a Reverse Transcription Kit (Qiagen, Redwood City, CA, USA). All Primers were purchased as DNA Oligo-Primer from Qiagen. Real-time PCR was performed on a Roche LightCycler480 Real-Time PCR System. The relative amount of each mRNA was calculated using the 2^−ΔΔCt^, which ΔΔCt =  ΔCt “treatment” − ΔCt “control.” The information of all primers used in this study is listed in Table 2.

### 3.2. Statistical Analysis

Data were analyzed in GraphPad Prism (version 8.4.3, San Diego, CA, USA) using unpaired Students *t*-test analysis and one or two-way ANOVA to compare each group. All numerical data were presented as mean ± SD. The *p*-value of < 0.05 was considered significant.

## 4. Results

### 4.1. Effects of Blue Light Irradiation on Cell Viability and Proliferation

**Figure 1:**
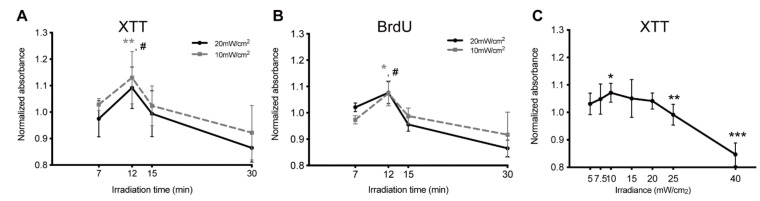
Blue light affects the cell viability of human umbilical vein endothelial cells (HUVECs) in a biphasic manner: (**A**) Changes in the cell viability of HUVECs following different blue light fluences with a constant time point of XTT assay at 24 h after irradiation. Absorbance was normalized to non-irradiated controls. Data are shown as mean ± SD (*n* = 3 repetitions, ** *p* < 0.01: 10 mW/cm^2^ vs. control, # *p* < 0.05: 20 mW/cm^2^ vs. control, Student *t*-test). (**B**) Changes in the cell proliferation of HUVECs following different blue light fluences. BrdU assays are shown 24 h after irradiation. Absorbance was normalized to non-irradiated controls. Data are shown as mean ± SD (*n* = 3 repetitions, * *p* < 0.05: 10 mW/cm^2^ vs. control, # *p* < 0.05: 20 mW/cm^2^ vs. control, Student *t*-test). (**C**) Changes in the cell viability of HUVECs following different blue light fluences with a constant irradiance and fixed time point 12 min of XTT assay at 24 h after irradiation. Absorbance was normalized to non-irradiated controls. Data are shown as mean ± SD (*n* = 3 repetitions, * *p* < 0.05, ** *p* < 0.01, *** *p* < 0.001 vs. control, Student *t*-test).

To understand the effect of blue light irradiation on HUVECs’ viability, cells were treated with two intensities, 10 mW/cm^2^ and 20 mW/cm^2^, for different time durations based on the irradiation parameters used in previous studies [23,24,25]. XTT assay results show that HUVECs responded biphasically to the blue light exposure. Irrespective of irradiances, short irradiation time increased the cell viability while treatment over a longer duration decreased the cell viability (Figure 1A). The highest cell viability was obtained with 12 min irradiation. Here, cells showed a 13% increase with 10 mW/cm^2^ as compared to control. A 9% rise was observed with 20 mW/cm^2^ as compared to control. The cell viability dropped to 92% (10 mW/cm^2^, irradiated group) and 86% (20 mW/cm^2^ irradiated group) compared to control after 30 min irradiation. The biphasic effect of blue light on HUVECs was also observed in the BrdU assays. Cell proliferation increased with irradiation for a short duration peaking at 12 min (7.6% and 7.4% compared to control, with 10 mW/cm^2^ and 20 mW/cm^2^, respectively), while cell proliferation was inhibited following longer irradiation times of more than 12 min (Figure 1A). At 15 min it decreased to 98% and 95% as compared to control, with 10 mW/cm^2^ and 20 mW/cm^2^, respectively (Figure 1B).

We further explored the influence of different irradiances on HUVECs. To this end, HUVECs were illuminated at different intensities for 12 min. Low intensities of blue light treatment promoted cell proliferation. The highest increase in cell viability (7.2% compared to control) was observed with 10 mW/cm^2^, while the cell viability decreased to 84.7% when cells were irradiated with 40 mW/cm^2^ (Figure 1C). Based on these results, two parameters were chosen for the following experiments: a low fluence group (10 mW/cm^2^ × 12 min, 7.2 J/cm^2^) and a high fluence group (40 mW/cm^2^ × 12 min, 28.8 J/cm^2^).

### 4.2. Effect of Blue Light on Cell Redox Change and Apoptosis

Blue light irradiation has been associated with increased intracellular ROS production [26,27], therefore we next evaluated ROS changes in HUVECs after blue light irradiation using the Grx1-roGFP3 redox sensor and DCFH-DA dye.

**Figure 2:**
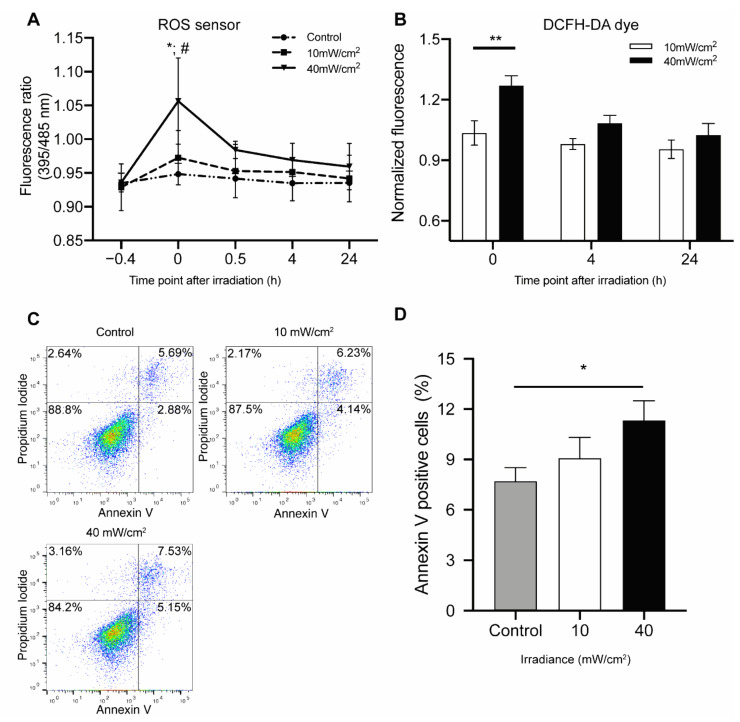
Blue light affects intracellular reactive oxygen species (ROS) production and apoptosis: (**A**) Dynamic ROS changes of HUVECs with redox sensor following blue light exposure to either low fluence (10 mW/cm^2^ × 12 min) or high fluence (40 mW/cm^2^ × 12 min). Data are shown as mean ± SD (*n* = 3 repetitions, * *p* < 0.05: 40 mW/cm^2^ vs. 10 mW/cm^2^, # *p* < 0.05: 40 mW/cm^2^ vs. control, two-way ANOVA) (**B**) Changes in the ROS levels in HUVECs following exposure to either low fluence (10 mW/cm^2^ × 12 min) or high fluence (40 mW/cm^2^ × 12 min), as evidenced by Dichloro-dihydro-fluorescein diacetate (DCFH-DA) assay after irradiation. Fold change was calculated versus non-irradiated controls. Data are shown as mean ± SD (*n* = 3 repetitions, ** *p* < 0.01, two-way ANOVA). (**C**) Representative results of Annexin V PI staining of HUVECs following exposure to either low fluence (10 mW/cm^2^ × 12 min) or high fluence (40 mW/cm^2^ × 12 min) measured by flow cytometry at 24 h after irradiation. (**D**) Quantification of cell apoptosis induced by blue light. Annexin V+/PI− and Annexin V+/PI+ populations were considered as apoptotic cells. Data are shown as mean ± SD (*n* = 3 repetitions. * *p* < 0.05).

A fluence-dependent increase in ROS level was observed immediately after blue light irradiation. High fluence illumination, i.e., 40 mW/cm^2^ × 12 min treatment, led to a 12% increase, while the low fluence irradiation, 10 mW/cm^2^ × 12 min, only led to a 4.9% ROS increase. The ROS levels returned to baseline 24 h after treatment (Figure 2A).

With DCFH-DA dye staining assays, we also found that blue light increased ROS production in HUVECs in a fluence-dependent manner (Figure 2B). High fluence irradiation increased ROS by 23.3% (*p* < 0.01) compared to low fluence irradiation. ROS levels returned to pre-irradiation level 24 h post-irradiation.

Increased ROS stress can lead to cell death. Therefore, we further evaluated if this blue light irradiation-mediated ROS increase led to apoptosis. Annexin V and PI staining was done on the HUVECs after irradiation. No apoptosis was observed after low fluence illumination and in the control group. Illumination at a high fluence slightly increased the number of apoptotic cells by 3.6% as compared to the control group (*p* < 0.05) (Figure 2C,D).

### 4.3. Effect of Blue Light on Cell Migration

**Figure 3:**
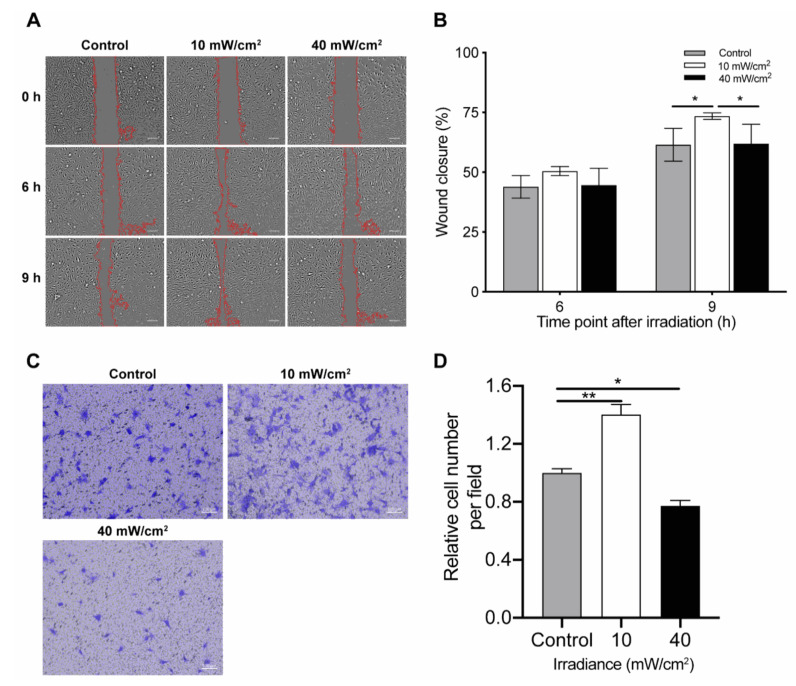
Blue light irradiation affected the cell migration of human umbilical vein endothelial cells (HUVECs): (**A**) Representative images of the wound healing assay of HUVECs following exposure to either low fluence (10 mW/cm^2^ × 12 min) or high fluence (40 mW/cm^2^ × 12 min) at 0, 6 and 9 h after irradiation. The scale bar is 100 μm. (**B**) Quantification of data from wound healing assays. Percentage of wound closure was measured as follows: Wound closure (%) = (original wound area—unhealed wound area)/original wound area) × 100%. Data are shown as mean ± SD (*n* = 3 repetitions, * *p* < 0.05, two-way ANOVA). (**C**) Representative images of the transwell assay of HUVECs following exposure to either low fluence (10 mW/cm^2^ × 12 min) or high fluence (40 mW/cm^2^ × 12 min) illumination at 6 h after irradiation. The scale bar is 100 μm. (**D**) Quantification of data from transwell assays. Data are normalized to control groups and shown as mean ± SD (*n* = 3 repetitions, * *p* < 0.05, ** *p* < 0.01, one-way ANOVA).

To explore whether blue light could also influence cell migration, wound healing assays were performed. Cells irradiated by a low fluence (10 mW/cm^2^ × 12 min) closed the wound significantly faster than the control cells after 9 h (*p* < 0.05, compared with the control group). In comparison to control non-irradiated cells, no significant differences were observed in cells irradiated by high fluence (40 mW/cm^2^ × 12 min) (Figure 3A,B).

These results were confirmed in the transwell assays, which showed 38% of cells migrated through the membranes 6 h after low fluence illumination in comparison to control cells (10 mW/cm^2^ × 12 min) (*p* < 0.01). Contrary to the findings of the wound healing assay, blue light irradiation with a high fluence inhibited the migration of cells (*p* < 0.05) (Figure 3C,D).

### 4.4. Effect of Blue Light on the Angiogenic Potential of HUVECs

**Figure 4:**
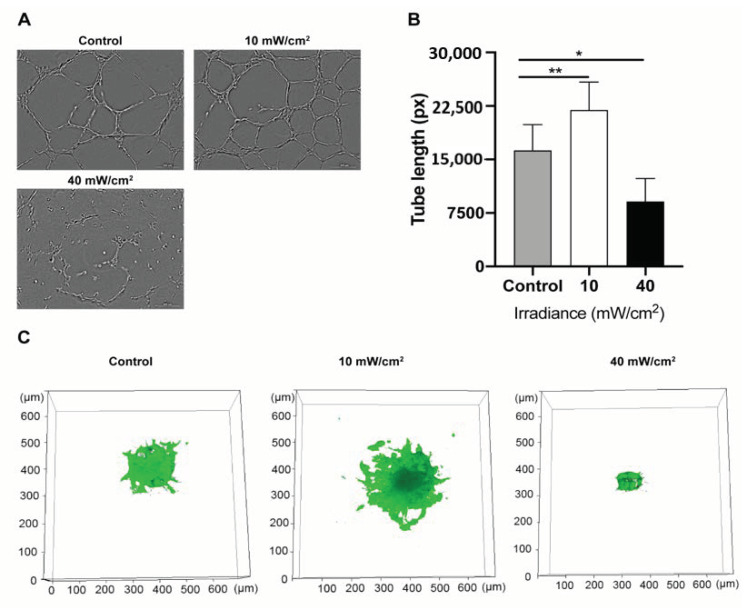
Blue light affected tube formation and sprouting in human umbilical vein endothelial cells (HUVECs): (**A**) Representative images of the tube formation assay of HUVECs following exposure to either low fluence (10 mW/cm^2^ × 12 min) or high fluence (40 mW/cm^2^ × 12 min) illumination at 6 h after irradiation. (**B**) Quantification of data from tube formation assays. Data are shown as mean ± SD (*n* = 3 repetitions, * *p* < 0.05, ** *p* < 0.01, one-way ANOVA). (**C**) Representative images of the spheroid sprouting assay of HUVECs with GFP following exposure to either low fluence (10 mW/cm^2^ × 12 min) or high fluence (40 mW/cm^2^ × 12 min) at 6 h after irradiation.

To analyze the effect of blue light irradiation on the angiogenic function of HUVECs, tube formation and spheroids sprouting assays were conducted. Longer tube-lengths formed 6 h after blue light treatment in the low fluence group (*p* < 0.01, in comparison to the non-irradiated group) (Figure 4A,B). Contrary to this, fewer tubes appeared in the high fluence group (*p* < 0.05 in comparison to the non-irradiated group) (Figure 4B). Likewise, in the sprouting assay, more sprouting area was observed in the low fluence treated group (*p* < 0.01 irradiated vs. non-irradiated control) while sprouting of HUVEC spheroids was inhibited by blue light irradiation at higher fluences (*p* < 0.01 irradiated vs. non-irradiated control) (Figure 4C and Figure S2).

### 4.5. Gene Expression Analysis from RNA-Sequencing

**Figure 5:**
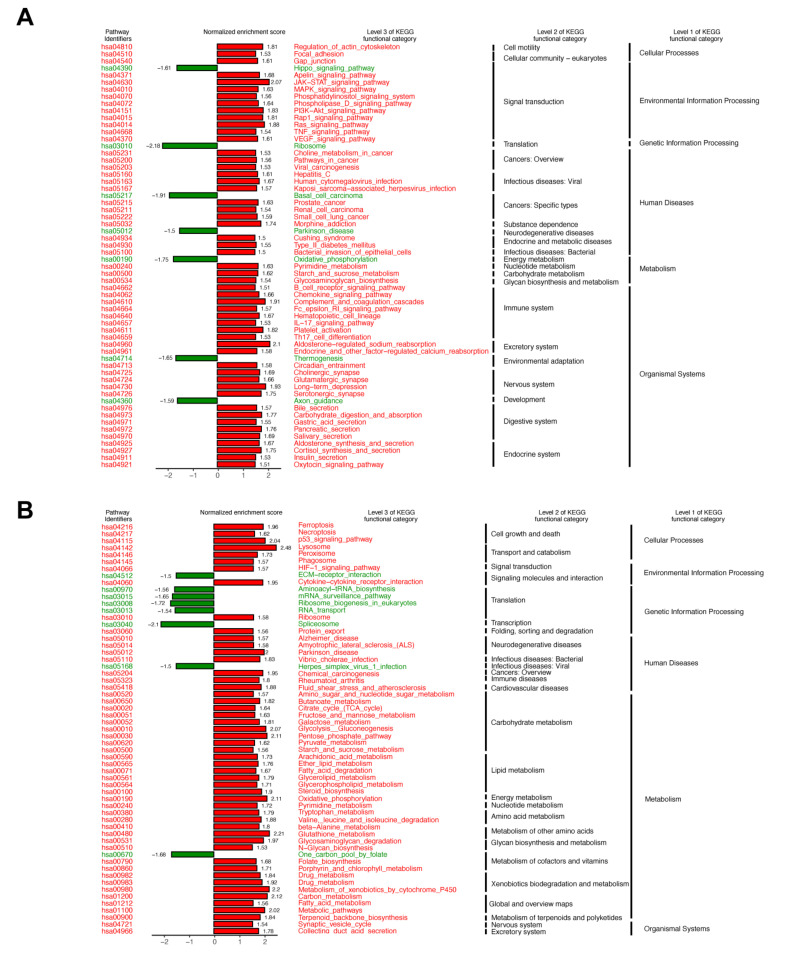
Kyoto Encyclopedia of Genes and Genomes (KEGG) pathway enrichment analysis of RNA sequencing: Significantly different functional KEGG categories (NES > 1.5 or <−1.5 and *p* < 0.05) were shown. (**A**) Results of low fluence (10 mW/cm^2^ × 12 min) versus the non-irradiated control group. (**B**) Results of high fluence (40 mW/cm^2^ × 12 min) versus the non-irradiated control group. NES: normalized enrichment score.

To study changes in the gene expression profiles of the HUVECs after blue light irradiation, RNA-sequencing was performed. The principal components analysis (PCA) showed three distinct clusters corresponding to non-irradiated control, low fluence and high fluence treatment (Figure S1). Differential gene expression analysis identified 409 genes of which 250 were upregulated. In the low fluence treatment, 159 genes were downregulated as compared to the control (Table S1). In the high fluence group vs. control (Table S1), 25 genes, of which 17 were upregulated and 8 genes which were downregulated, were observed The KEGG enrichment analysis revealed that the most affected pathways (normalized enrichment score (NES) > 1.5 or NES < −1.5) were upregulated in the low fluence group compared to non-irradiated control. The main category was the “organismal systems,” and the subcategory was the “signal transduction” including the mitogen-activated protein kinase (MAPK) signaling pathway, the Janus kinases (JAK)- signal transducer and activator of transcription proteins (STAT) signal pathway, and the phosphatidylinositol 3-kinases—Protein kinase B (PI3K-Akt/PKB) pathway (Figure 5A).

**Figure 6:**
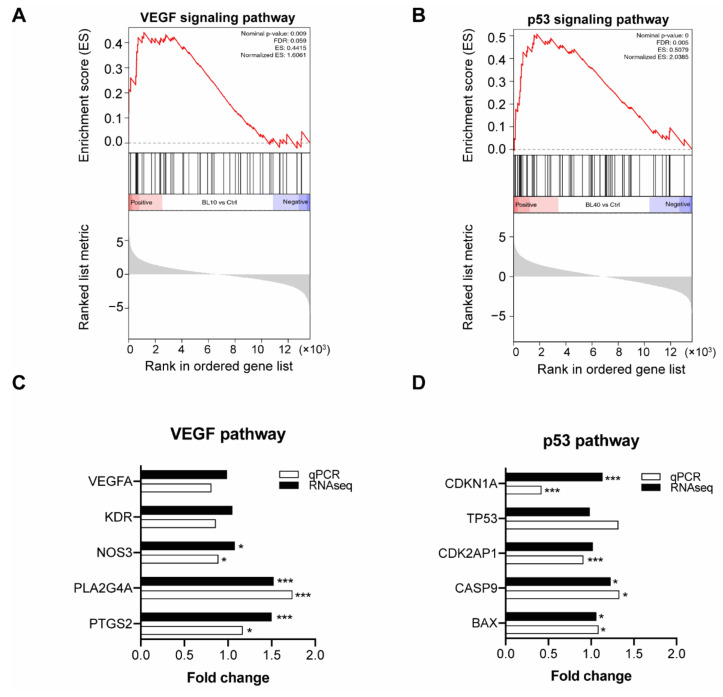
Gene set enrichment analysis (GSEA) and qPCR validation: (**A**) Vascular endothelial growth factor (VEGF) signaling pathway was upregulated in the low fluence (10 mW/cm^2^ × 12 min) group. (**B**) p53 signaling pathway was upregulated in the high fluence (40 mW/cm^2^ × 12 min) group. (**C**) qPCR validation of genes in the VEGF signaling pathway and comparison with RNA-seq data. Fold change was normalized with control (*n* = 3 repetitions, * *p* < 0.05, *** *p* < 0.001, Student *t*-test). (**D**) qPCR validation of genes in the p53 signaling pathway and comparison with RNA-seq data. Fold change was normalized with control (* *p* < 0.05, *** *p* < 0.001, Student *t*-test).

“Metabolism” was the most regulated main category in cells irradiated at high fluences—especially the carbohydrate metabolism (Figure 5B). Furthermore, we also found enrichment of the vascular endothelial growth factor (VEGF) pathway (NSE = 1.61) in the low fluence group while in the high fluence group, the p53 pathway was enriched (NES = 2.04) (Figure 6A,B).

### 4.6. Real-Time PCR Verifies Genes in Enriched Pathways

Real-time PCR validation of RNA sequencing data was performed for selected genes in the enriched VEGF and p53 pathway. The validation was performed with RNA samples derived 24 h after blue light irradiation. Real-time PCR results partially matched the RNA sequencing results. We found in the VEGF pathway two genes enriched in the low fluence group; PLA2G4A and PTGS2 were highly expressed after low fluence blue light irradiation. In the enriched p53 pathway of the high fluence group, CASP9 and BAX were significantly upregulated (Figure 6C,D).

## 5. Discussion

In this study, we systematically investigated the impact of LED blue light with a wavelength of 453 nm on HUVECs. We demonstrated firstly that blue light irradiation regulates the biological activities of HUVECs in vitro and yields a biphasic effect. On the one hand, at a low fluence (10 mW/cm^2^ × 12 min, 7.2 J/cm^2^), blue light promoted cell viability, migration, and angiogenic function of HUVECs. On the other hand, at high fluence (40 mW/cm^2^ × 12 min, 28.8 J/cm^2^) blue light irradiation inhibited all the above-mentioned cell activities. Secondly, we showed that blue light increased intracellular ROS production in a fluence-dependent manner. Thirdly, we revealed the mechanisms of the observed biphasic effect by RNA sequencing. Several pathways, e.g., MAPK, JAK-STAT, PI3K-Akt and VEGF—known to improve cell viability, migration, and angiogenesis—were upregulated in the low fluence group, while ferroptosis, necroptosis and the p53 signaling pathways—which are known to negatively regulate above cell activities—were activated after higher fluences of light irradiation.

The similar biphasic effects of blue light on HUVECs were demonstrated in other wavelengths of low-level light therapy. For instance, laser irradiation (810 nm) at 6 mW/cm^2^ × for 3 days showed the most significant reduction in rat (62%) and dog (49%) myocardial infarction, while high irradiation at 20 mW/cm^2^ achieved less effect (in rat 2.8%) [28]. Furthermore, a similar trend was found in the wound healing of mouse pressure ulcer model when different doses of light at 670 nm were applied [29]. This light dose-dependence could be explained by the “Arndt-Schulz Law”, which states that weak stimuli slightly promote biological activities until the peak is reached, and then stronger stimuli start to induce suppression [30].

With a low fluence of irradiation (10 mW/cm^2^ × 12 min, 7.2 J/cm^2^), blue light (453 nm) promoted cell viability, migration, and sprouting of HUVECs. These findings are well-supported with the upregulation of the angiogenesis-related pathways in RNA sequencing analysis, especially VEGF pathway which plays a central role in angiogenesis. Furthermore, expression of several genes in the VEGF pathway were significantly increased. The first gene, PTGS2, also known as cyclooxygenase−2 (COX-2), has been reported to be involved in the VEGF-mediated angiogenic potential of HUVECs through activating the p38 MAKP and JNK kinase pathways [31]. Interestingly, we also found these pathways to be upregulated in the KEGG enrichment analysis. The second upregulated gene, PLA2G4A (alias for cPLA2 gene), had no direct connection with blue light or angiogenesis. So far, only one study has indicated that cPLA2 was regulated by MAP kinase [32], which was found upregulated after low fluence irradiation. In concordance with our findings, several in vivo studies have revealed a potential positive regulation of angiogenesis by blue light. For example, in rats, the wound healing of the abdominal flap was accelerated by a blue light stimulus with 50 mW/cm^2^ × 10 min/per day (470 nm) by enhancing the local angiogenesis [11]. Furthermore, a recent clinical study showed that 30 min of blue light (453 nm, 42 mW/cm^2^) treatment decreased the blood pressure by increasing NO releasing into circulating blood in healthy volunteers [12]. Several other studies have also focused on the role of nitric oxide production (NO) under blue light illumination, as NO plays a vital role in maintaining vascular homeostasis [33,34]. One study showed blue light (420–453 nm) induced NO formation in human skin in vitro and in vivo [35]. In our study, we also found the upregulation of nitric oxide synthase 3 (NOS3) using RNA sequencing and qPCR analysis. In summary, the effects of blue light at low fluences on cell viability, cell migration, and angiogenesis could be mainly attributed to the activation of angiogenesis-related pathways.

Although our study used blue light irradiation at 453 nm, a wavelength described as less harmful for cells [36,37], we also observed inhibitory effects on cell viability and migration, and an increase in cell apoptosis using blue light at a higher fluence (40 mW/cm^2^ × 12 min, 28.8 J/cm^2^), which is consistent with the results observed in several types of cells. For instance, one study showed that blue light with 453 nm at a fluence of 33 J/cm^2^ was not toxic to human keratinocytes and skin-derived endothelial cells compared to wavelengths of 412–426 nm at high fluences (66–100 J/cm^2^), but it would reduce the cell proliferation, while wavelengths of 632–940 nm had no effect on the growth of these two cell types [36]. The reduction of cell growth induced by blue light at 453 nm was reversed by adding vitamin C. This result indicated that radical species play a role in the blue light-regulated cell activities. Similarly, cell growth of the retinal pigment epithelial cells, aortic endothelial cells and fibroblasts were reported to decrease after exposure to 42 J/cm^2^ of blue light irradiation (wavelength from 425–500 nm), and the red (580–700 nm) or green (495–650 nm) light as well did not affect cell growth [14]. Inhibition of migration and invasion properties of colon cancer cell lines upon irradiation with LED blue light (450 nm, 6.3 mW/cm^2^ for 30 min) was also reported [38]. Up to now, there is limited data on the effect of blue light irradiation on HUVECs. Only one study describes that blue light (470 nm) used at a fluence of 24 J/cm^2^ slightly decreased the migration of HUVECs in wound healing assays [13].

This inhibitiory effect of blue light irradiation on HUVECs may be mainly attributed to the induction of ROS production, as many studies revealed that blue light irradiation could increase ROS generation and influence cell’s redox status [30]. For instance, blue LED light (470 nm) at 20 mW/cm^2^ induced ROS production in bone marrow-derived mesenchymal stem cells in a dose-dependent manner, which further caused cell apoptosis [39]. In retinal pigment epithelium cells, blue light (440 ± 10 nm) at 4 mW/cm^2^ could induce cell death through a free-radical-associated mechanism [40]. Our previous study of immortalized human keratinocytes (HaCaT) also revealed that blue light with a fluence of 41.35 J/cm^2^ induced an increase of the intracellular H_2_O_2_ shortly after irradiation [23]. This rapid increase declined after 24 h. In our study, we found a similar trend in HUVECs. Furthermore, we saw a mild boost of apoptosis in HUVECs after high fluence irradiation. Excess amounts of cellular ROS leads to oxidation of proteins, nucleic acids, lipids, membranes and organelles, which can lead to an activation of programmed cell death [30,41]. KEGG pathway enrichment analysis of RNA sequencing data from cells exposed to high fluences of blue light reveals upregulation of ferroptosis, necroptosis and p53 pathways. Upregulation of these pathways due to high cellular stress might be another potential underlying mechanism behind the induction of ROS production and apoptosis by blue light in HUVECs. qPCR validation of the p53 pathway showed that CASP9 and BAX were significantly upregulated in cells irradiated by high fluence blue light. CASP9 encodes the Caspase-9 protease required for apoptosis [42,43] and BAX is an apoptosis regulator involved in the p53-regulated apoptosis [44,45]. The induction of blue light-mediated apoptosis may also explain the changes observed for the other cell behavior in our study (cell viability, migration, and angiogenesis).

Compared with other wavelengths of light, blue light demonstrated dominant biological functions from quite a number of studies, in which blue light showed bactericidal effects [46,47] and anti-inflammatory properties [6,7]. This indicates that infected wounds would be the optimal choice for blue light treatment. Combined with our findings of blue light in angiogenesis, blue light could be applied for enhancing local angiogenesis at the same time. The inhibitory part of blue light may be used for the treatment of hemangioma or melanoma by suppressing or even reducing angiogenesis. However, the right dose of blue light treatment should be explored as overdose of light treatment would cause cell death. The penetration of blue light should be taken into account before any in vivo application. Additionally, the parameters such as irradiance or time used in the present study have to be confirmed or modified in other types of ECs and in vivo experiments, as these irradiation parameters are key factors to the cell response [30].

## 6. Conclusions

Blue light irradiation at 453 nm biphasically regulated the cell activities of HUVECs. With low fluence light treatment, cell viability, migration, and angiogenesis were promoted by activating the angiogenic VEGF pathway, which may explain the role of blue light in promoting wound healing. In contrast, high fluence blue light irradiation showed the opposite effect on the above cell activities. This inhibition was mediated through the upregulation of apoptosis and the p53 signaling pathway. Therefore, these findings may be a useful strategy for the regulation of angiogenesis.

## Figures and Tables

**Table 1:** Preparation of the amplifying hydrogel solution (AHS).

Solution Components	Concentration	Manufacturer
40% acrylamide	20%	Bio-Rad, Munich, Germany
2% bis-acrylamide	0.05%	Bio-Rad, Munich, Germany
16% PFA	4%	Electron Microscopy Sciences, Hatfield, USA
Sodium acrylate	10%	Sigma-Aldrich, Munich, Germany
10% VA-044	0.1%	FUJIFILM, Osaka, Japan
10× PBS	1×	Sigma-Aldrich, Munich, Germany

**Table 2:** Primers used in the study.

Gene Symbol	Qiagen Category Number
VEGFA	QT01010184
KDR	QT00069818
NOS3	QT00089033
PLA2G4A	QT00085813
PTGS2	QT00040586
CDKN1A	QT00044233
TP53	QT02377634
CDK2AP1	QT00226198
CASP9	QT00036267
BAX	QT00031192
GAPDH	QT01192646

## Data Availability

The data presented in this study are available on request from the corresponding author.

## Supplementary Materials

The following are available online at https://www.mdpi.com/article/10.3390/biomedicines9070829/s1, Figure S1: Principal component analysis (PCA) and cluster analysis of RNA-seq data. Figure S2: Quantification of data from spheroid sprouting assay. Table S1: Results of differentially expressed genes.
