# Supplementary figures and images for: Biphasic Effects of Blue Light Irradiation on Human Umbilical Vein Endothelial Cells
Source: Biomedicines. 2021 Jul 16;9(7):829. doi: 10.3390/biomedicines9070829 (PMC8301484; doi:10.3390/biomedicines9070829)

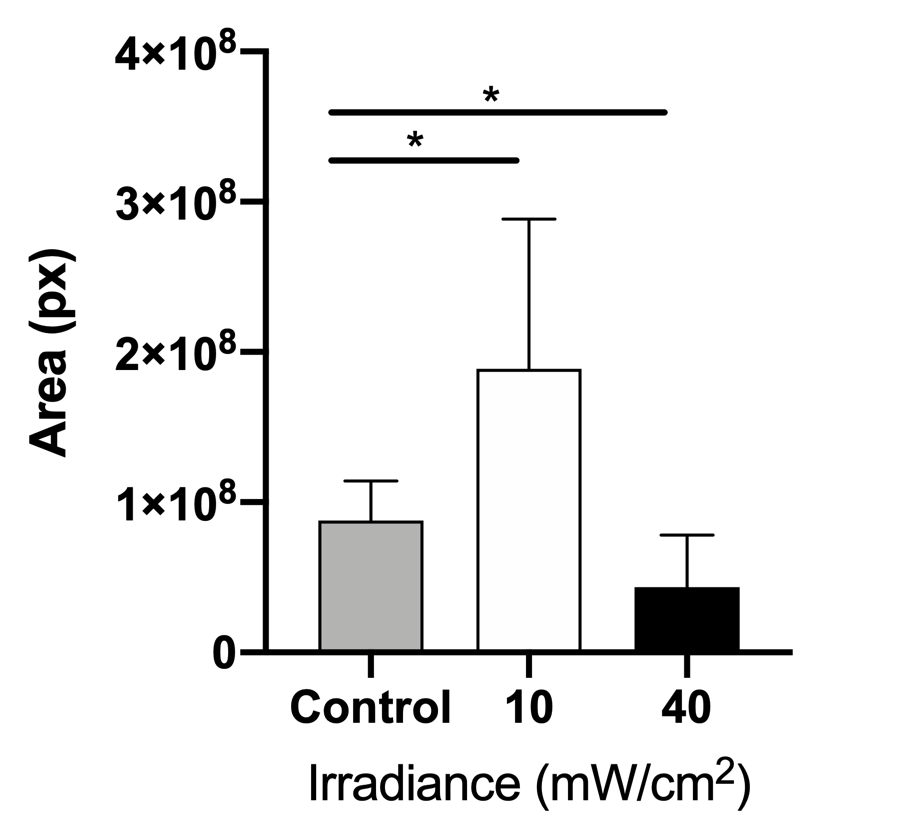

Supplement: Supplementary file 1 [file biomedicines-09-00829-s001.zip › supplement/FS2.tiff]

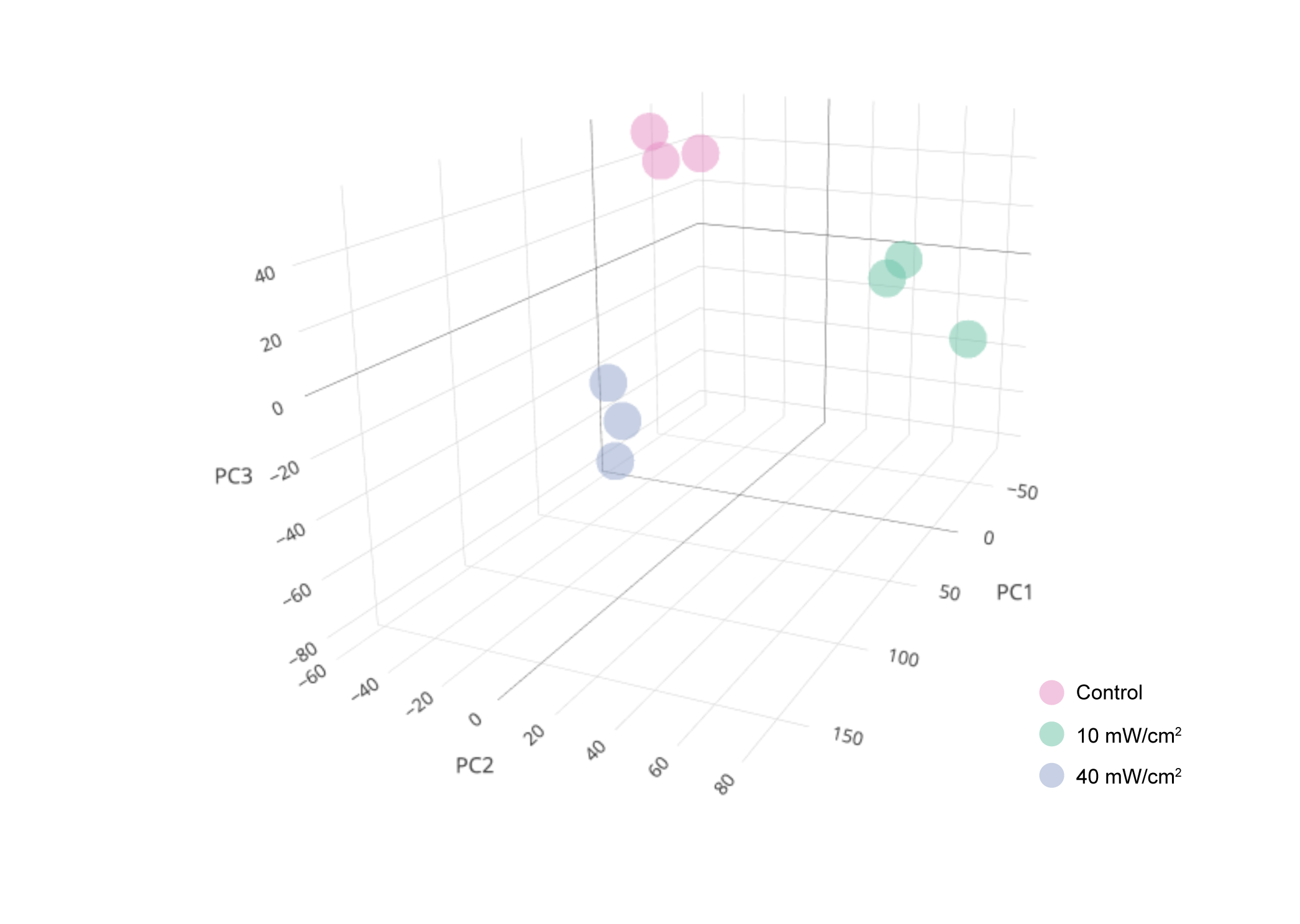

Supplement: Supplementary file 1 [file biomedicines-09-00829-s001.zip › supplement/FS1.tif]
